# Thirty-second sit-to-stand test as an alternative for estimating peak oxygen uptake and 6-min walking distance in women with breast cancer: a cross-sectional study

**DOI:** 10.1007/s00520-022-07268-z

**Published:** 2022-07-11

**Authors:** Estíbaliz Díaz-Balboa, Violeta González-Salvado, Beatriz Rodríguez-Romero, Amparo Martínez-Monzonís, Milagros Pedreira-Pérez, Antonio I. Cuesta-Vargas, Rafael López-López, José R. González-Juanatey, Carlos Pena-Gil

**Affiliations:** 1 Department of Physiotherapy, Medicine and Biomedical Sciences, University of A Coruna, Campus de Oza, 15071 A Coruña, Spain; 2 Cardiology Department, Centro de Investigación Biomédica en Red de Enfermedades Cardiovasculares (CIBERCV), University Clinical Hospital of Santiago de Compostela (SERGAS), 15706 Santiago de Compostela, A Coruña, Spain; 3 Health Research Institute of Santiago de Compostela (IDIS), 15706 Santiago de Compostela, A Coruña, Spain; 4 Instituto de Investigación Biomédica de Málaga (IBIMA), 29010 Malaga, Spain; 5 Department of Physiotherapy, University of Málaga, 29071 Malaga, Spain; 6 School of Clinical Sciences of the Faculty of Health, Queensland University of Technology, Brisbane, 4000 Australia; 7 Medical Oncology Department and Translational Medical Oncology Group, Centro de Investigación Biomédica en Red de Cáncer (CIBERONC), University Clinical Hospital of Santiago (SERGAS), Santiago de Compostela University School of Medicine, 15706 Santiago de Compostela, A Coruña, Spain

**Keywords:** Breast cancer, Peak oxygen uptake, Six-minute walking distance, Sit-to-stand test, Cardio-oncology rehabilitation

## Abstract

**Purpose:**

To determine whether the 30-s sit-to-stand (30STS) test can be a valid tool for estimating and stratifying peak oxygen uptake (VO2peak) and 6-min walking distance (6MWD) in women with breast cancer.

**Methods:**

This cross-sectional study uses data from the ONCORE randomized controlled trial, including 120 women aged 18–70 years with early-stage breast cancer under treatment with anthracycline and/or anti-HER2 antibodies. Participant characteristics were collected at baseline and pooled data from functional assessment (30STS test, relative and absolute VO2peak, and 6MWD) were collected at baseline and post-intervention (comprehensive cardio-oncology rehabilitation program vs. usual care). Bivariate correlations and multivariate linear regression analyses were performed to study the relationship between functional test variables.

**Results:**

The number of repetitions in the 30STS test showed (i) a moderate correlation with relative VO2peak (ml/kg/min) (*r* = 0.419; *p* < 0.001; *n* = 126), (ii) a weak correlation with absolute VO2peak (ml/min) (*r* = 0.241; *p* = 0.008; *n* = 120), and (iii) a moderate correlation with the 6MWD (*r* = 0.440; *p* < 0.001; *n* = 85). The ONCORE equations obtained from the multivariate regression models allowed the estimation of VO2peak and 6MWD (*r*^*2*^ = 0.390; *r*^*2*^ = 0.261, respectively) based on the 30STS test, and its stratification into tertiles (low, moderate, and high).

**Conclusion:**

The 30STS test was found to be a useful tool to estimate VO2peak and/or 6MWD in women with early-stage breast cancer. Its use may facilitate the assessment and stratification of functional capacity in this population for the implementation of therapeutic exercise programs if cardiopulmonary exercise testing (CPET) or 6MWT are not available.

**Trial registration:**

ClinicalTrials.gov Identifier: NCT03964142. Registered on 28 May 2019. Retrospectively registered. https://clinicaltrials.gov/ct2/show/NCT03964142

## Introduction

Breast cancer treatment impacts on patients’ physical performance, decreasing cardiopulmonary function [1], as well as muscle strength, affecting their activities of daily living [2]. The benefits and safety of exercise in maintaining or improving exercise tolerance and mitigating side effects are well known in breast cancer patients [3, 4]. Consequently, the implementation of programs that include therapeutic exercise, such as Cardio-Oncology Rehabilitation (CORe) programs, is emerging as complementary therapeutic tools to improve the patient’s quality of life [5]. Clinical assessment and stratification of functional capacity of patients with breast cancer are the first necessary steps for adequate patient screening and prescription of therapeutic exercise programs, in order to optimize treatment outcomes [6].

Different tests can be used for this purpose, the measurement of peak oxygen uptake (VO2peak) being the gold standard for assessing cardiopulmonary function, as it is the most important determinant of general health. VO2peak is considered a powerful and independent predictor of cardiovascular risk, stronger than classical risk factors [7]. Moreover, it has been reported that individuals with a high level of cardiopulmonary function have a 45% lower risk of total cancer mortality compared to those with low cardiopulmonary function, regardless of adiposity [8]. Currently, VO2peak reference standards for apparently healthy adults obtained from cardiopulmonary exercise testing (CPET) are available, derived from the Fitness Registry and the Importance of Exercise National Database (FRIEND) [9]. However, in patients with breast cancer, VO2peak may decrease between 5 and 26% during exposure to different treatments [1]. Additionally, VO2peak was found to be significantly reduced in breast cancer patients prior to adjuvant therapy as compared to healthy sedentary women by 17%, a difference that increased up to 25% after completing treatment [10]. This may suggest that normative VO2peak values for women may not be representative to women with breast cancer [10].

CPET is the most objective and accurate test for direct determination of VO2peak [11]. However, it may not be accessible in all clinical settings. Alternatively, the 6-min walk test (6MWT) is used for the assessment of functional exercise capacity in numerous pathologies, as it is a more accessible test [12, 13]. The 6MWT is developed over a 6-min walking on a flat and hard surface [14], including measurements of distance (6MWD, in meters), heart rate, fatigue, perceived exertion, oxygen saturation, walking speed, or stride length [15]. The 6MWT was originally performed on patients with cardiopulmonary disease [12]. However, it is being widely used in the assessment of breast cancer patients [16, 17], showing positive and strong correlation with VO2peak (*r* = 0.67) and a good reliability (intraclass correlation coefficient, ICC = 0.93) and validity (coefficient of variation, CV = 3%) [18].

Another useful tool to estimate cardiopulmonary function may be the sit-to-stand (STS) test, which is based on a mechanically demanding movement of daily life involving large muscle groups from the legs and trunk [19]. It has the advantage of requiring little time and material and can be easily performed in most clinical settings, and has shown strong association with 1-Maximal Repetition leg press (*r* = 0.68, *p* < 0.05) and a good reliability (ICC = 0.80) in healthy subjects [20]. The STS test has traditionally been used in the assessment of lower limb strength in elderly people [21] and has now extended to other clinical populations such as breast cancer patients [22], where it has also been used as an anaerobic lactic stress test [23]. Although the usefulness of the STS test for estimating cardiopulmonary function has been studied in other clinical populations [24], it has barely been investigated in women with breast cancer.

Considering the importance of having more accessible and cost-effective tests for estimating cardiopulmonary function and facilitating the implementation of oncological exercise programs, the objectives of this study, performed with a cohort of women with breast cancer at early stages receiving neoadjuvant/adjuvant chemotherapy, are as follows: (1) to determine the relationship between 30STS test and VO2peak derived from CPET, and to develop an equation for estimating and stratifying VO2peak based on a 30STS test and (2) to determine the relationship between 30STS test and 6MWD, and to develop an equation for estimating and stratifying 6MWD based on the 30STS test. It was hypothesized that there would be a strong association between the 30STS test with VO2peak and with the 6MWD, as these tests are based on global movement with significant metabolic demand and involve large muscle groups from lower limbs and trunk. This could allow the estimation and stratification of cardiopulmonary function by performing a simple 30STS test.

## Materials and methods

### Study setting and design

This inferential cross-sectional study has been carried out with data from the ONCORE study, of which its protocol — including modifications due to the COVID-19 pandemic — has been previously published [25] and reported in the ClinicalTrials database (NCT03964142). The ONCORE study is a two-arm, prospective, randomized controlled trial (RCT) comparing the effectiveness of a comprehensive CORe program (experimental group) to prevent cardiotoxicity in women with non-metastatic breast cancer receiving anthracycline and/or anti-HER2 antibodies, as compared to usual care and physical activity recommendation (control group). Although patients are randomly assigned after the first assessment to the experimental or the control arm, pooled data from all participants were used to conduct this investigation, with no distinction between groups. The trial is being developed at the University Clinical Hospital of Santiago de Compostela (Spain) and has been approved by the Ethics Committee of Clinical Investigation of Galicia (2018/083). The trial is scheduled to conclude in March 2022, when the last patient included will have completed her chemotherapy treatment and undergone the post-intervention assessment.

### Participants

For the purpose of the present study, we collected data from 120 women aged 18–70 years with a first diagnosis of early-stage breast cancer (I, II, III) who received anthracycline and/or anti-HER2 antibodies and were included in the ONCORE study between August 2018 and March 2021. Inclusion and exclusion criteria have been detailed in the ONCORE study protocol [25]. Coordinated management of participants includes cancer treatment and supervision by the Oncology department, cardiotoxicity monitoring by the Cardio-Oncology unit, and functional assessment by the Cardiac Rehabilitation unit before and after the CORe program, which lasts as long as the cardiotoxic chemotherapy.

### Measurements

#### Anthropometric, vital signs, and breast cancer characteristics

Height (m), weight (kg), body mass index (as kg/m^2^), and abdominal circumference (cm) were measured. Vital signs, including heart rate (bpm) and blood pressure (mmHg), were assessed with a blood pressure monitor (Omron M3 IT). The characteristics of the oncological process — disease stage, molecular subtype, and chemotherapy regimen and timing — were obtained from the patient’s electronic medical records.

#### Thirty-second sit-to-stand

The 30STS test was performed on a 44-cm chair stabilized against a wall to prevent displacement [21]. To conduct the test, the patient stood in front of the chair in a standing position with feet pelvis-width apart and arms crossed, with hands at shoulders. The patient was asked to perform as many repetitions as possible in 30 s, considering only those in which she touched the chair with the thighs or buttocks and returned to the initial position by extending the knees and hips. A demonstration was performed beforehand by the evaluator. The total number of completed repetitions was recorded.

#### Cardiopulmonary exercise test

CPET with 12-lead electrocardiographic monitoring was performed by a cardiologist and a physiotherapist to determine VO2peak on a continuous incremental test in a cycle ergometer (CardioWise Ergo Fit, Pirmasens, Germany) with breath-by-breath analyzer (MS-CPX/SBx/CPx, Jaeger, Cardinal Health Germany). Relative (ml/kg/min) and absolute (ml/min) values of VO2peak were considered. After performing spirometry and before the stress test, an individualized continuous ramp protocol was selected for each patient considering the CPET software suggestion according to patient’s characteristics, and also the clinician’s criteria, to achieve an exercise duration of 8–12 min [26] and/or voluntary exhaustion. A 3-min resting period was followed by a 3-min warm-up period with continuous pedaling at 5 W. Workload was increased by 9 or 12 W/min until patient’s exhaustion, appearance of other limiting symptoms, or the presence of risk alerts as determined by the cardiologist. Patients maintained a cadence of 65 revolutions/min, the minimum admissible value being 60 rpm. During exercise, blood pressure was recorded every 3 min with a standing blood pressure monitor (Tango M2, SunTech Medical, USA) and oxygen saturation was determined by pulse oximetry. Perceived effort was assessed using the modified Borg scale before and immediately after the test [27].

#### Six-minute walking test

The 6MWT was performed in a 30-m hallway [12]. In this test, the patient was asked to walk at maximum speed to achieve as many meters as possible in 6 min. The participant was informed of the time remaining with standardized phrases. The absolute value in meters for covered distance was recorded at the end of the test.

### Data management

The timing of the measurements performed, according to the development of the ONCORE study, is specified in this section. Participant characteristics were collected at baseline. Pooled data from functional assessment (30STS test, CPET, and 6MWT) were collected both at baseline and post-intervention. Due to the required adaptation of the study caused by the COVID-19 pandemic, when disinfection of ventilation systems of CPET could not be ensured and they were temporary cancelled at the hospital for safety concerns, the 6MWT was selected as an alternative to measure cardiopulmonary function. Consequently, participants performed either 30STS test and CPET, or 30STS test and 6MWT. In both alternatives, the two tests were done on the same day, with an interval of at least 10 min between each test, the 30STS test being performed first. Table 1 specifies the number and timing of the functional tests performed. All functional assessments were performed by the same trained investigator.

**Table 1:** Number and timing of functional tests performed and included in the analysis

Type of functional test performed	Baseline assessment	Post-intervention assessment	Pooled measurements
*30STS test and CPET*	*n* = 79	*n* = 47	126*
*COVID-19 PANDEMIC (6MWT instead of CPET)*			
*30STS test and 6MWT*	*n* = 41	*n* = 44	85

*30STS*, 30-s sit-to-stand test; *CPET*, cardiopulmonary exercise testing; *6MWT*, 6-min walking test

*Relative (ml/kg/min) and absolute (ml/min) VO2peak values were collected. Absolute VO2peak from 6 patients could not be obtained due to technical problems

### Statistical analysis

The descriptive analysis of patient characteristics included anthropometric characteristics, vital signs, breast cancer variables, and the functional tests used to estimate cardiopulmonary function. Continuous variables were presented as mean ± standard deviation (SD) and categorical variables were presented as numeric values (%). The normality of the distribution was tested with the Shapiro–Wilk test. Pearson’s correlation coefficient (*r*) was used to calculate the strength of the bivariable relation between functional test variables. To label the strength of the association for absolute values of *r*, 0–0.19 was regarded as very weak, 0.2–0.39 as weak, 0.40–0.59 as moderate, 0.6–0.79 as strong, and 0.8–1 as very strong [28]. Three regression models were calculated using multivariate linear regression analysis. As result of these analyses, two equations were provided (ONCORE equations) to estimate and stratify VO2peak and 6MWD from the repetitions of 30STS test. The statistical analysis was performed with SPSS version 25.

## Results

### Baseline participant characteristics

Baseline characteristics of the 120 participants are shown in Table 2. Mean age was 48.78 ± 8.23 years, mean BMI fell within the overweight range (26.56 ± 5.42), and the predominant cardiovascular risk factor was hyperlipidemia (37.5%). Patients received both adjuvant (40%) and neoadjuvant (60%) chemotherapy. Regarding functional assessment, mean relative VO2peak was 20.83 ± 4.26 ml/min/kg, mean absolute VO2peak was 1415.82 ± 256.34 ml/min, mean 6MWD was 606.1 ± 69.96 m, and mean number of repetitions in the 30STS was 20.03 ± 4.38.

**Table 2:** Baseline participant characteristics

Characteristic* (*n* = 120)	Mean ± SD/ *n*(%)**	Min–max
Age, years	48.78 ± 8.23	30–69
Height, cm	161.18 ± 5.64	148–178
Weight, kg	69.01 ± 13.61	45–121
BMI, kg/m^2^	26.56 ± 5.42	17.96–44.04
Waist perimeter, cm	88.98 ± 12.85	64–131
Menopausal status		
Pre-menopausal	76 (63.3%)	
Post-menopausal	44 (36.7%)	
Classic cardiovascular risk factors		
Arterial hypertension	10 (8.3%)	
Hyperlipidemia	45 (37.5%)	
Diabetes	5 (4.2%)	
Smoker	12 (9.8%)	
Ex-smoker	46 (38.3)	
Disease stage		
I	28 (23.3%)	
II	63 (52.5%)	
III	29 (24.2%)	
Molecular subtype		
Luminal A	30 (25%)	
Luminal B HER2-	31 (25.8%)	
Luminal B HER2 +	31 (25.8%)	
Pure HER2	10 (8.3%)	
Triple-negative	17 (14.2%)	
Chemotherapy		
Neoadjuvant	72 (60%)	
Adjuvant	48 (40%)	
Cycles of anthracyclines or AntiHER2 received at baseline assessment		
0 cycles	70 (58.3%)	
1 cycle	42 (35%)	
2 cycles	8 (6.7%)	
Functional assessment		
VO2peak of CPET, ml/kg/min (*N* = 79)	20.83 ± 4.26	13–30
VO2peak of CPET, ml/min (*N* = 76)	1415.82 ± 256.34	832–2096
Distance of 6MWT, m (*N* = 41)	606.1 ± 69.96	479–825
Repetitions of 30STS (*N* = 120)	20.03 ± 4.38	9–32

*CPET*, cardiopulmonary exercise test; *6MWT*, 6-min walking test

*The present characteristics correspond to the baseline of the ONCORE study

**Continuous variables are presented as mean ± standard deviation (SD) and categorical variables are presented as *n* (%)

### Correlation and multivariate regression analysis between functional test variables

The bivariate correlations (Pearson *r*) among functional tests were explored. The strength of the association between the 30STS test and VO2peak (relative and absolute) from CPET, and between the 30STS test and 6MWD was found to be as follows:

1. The number of repetitions from the 30STS test showed a significant, moderate correlation with relative VO2peak (ml/kg/min) (*r* = 0.419; *p* < 0.001; *n* = 126).
2. The number of repetitions from the 30STS test showed a significant, weak correlation with absolute VO2peak (ml/min) (*r* = 0.241; *p* = 0.008; *n* = 120).
3. The number of repetitions from the 30STS test showed a significant, moderate correlation with 6MWD (m) (*r* = 0.440; *p* < 0.001; *n* = 85).

The regression models are summarized in Table 3. Although the 3 models were significant (*p* < 0.001), model #1 was the best fitted. In fact, it was the most clinically relevant, as it could allow estimating VO2peak (ml/kg/min) using the number of repetitions from the 30STS test and the patient’s weight.

**Table 3:** Decomposition of the multivariate regression models

Model	Dependent variable	Independent variables	*B*	*β*	*r*	*r*^2^	SEE	*F*	Model *p* value
*#1*	Relative VO2peak (ml/kg/min)	Intercept	22.610		0.624	0.390	3.395	39.304	< 0.001
		30STS (reps)	0.347	0.338**					
		Weight (kg)	− 0.127	− 0.470**					
*#2*	Absolute VO2peak (ml/min)	Intercept	266.425		0.573	0.328	229.624	28.584	< 0.001
		30STS (reps)	24.429	0.361**					
		Weight (kg)	9.224	0.533**					
*#3*	6MWT (m)	Intercept	614.855		0.511	0.261	54.019	14.487	< 0.001
		30STS (reps)	5.150	0.384**					
		Age (years)	− 2.238	− 0.265*					

*B*, unstandardized coefficient; *β*, standardized coefficient; *r*, correlation coefficient; *r*^*2*^, coefficient of determination, *F*, *F*-test of the equality of two variances; *SEE*, standard error of the estimate; *30STS*, 30-s sit-to-stand; *VO2peak*, peak oxygen uptake; *6MWT*, 6-min walking test

*p<0.01; **p<0.001

### Estimation and stratification of VO2peak by the ONCORE equation

As a result of the previous regression model #1, the ONCORE equation was developed to allow clinicians to estimate VO2peak in women with breast cancer using the repetitions from the 30STS test and weight (kg). Consequently, the — *Estimated VO2peak —* formula would stand as follows:

$$\mathrm{Estimated}\;\mathrm{VO}2\;\mathrm{peak}\;(\mathrm{ml}/\mathrm{kg}/\min)=22.610404+\lbrack\mathrm{weight}\;{(\mathrm{kg})}^\ast-0.12736\rbrack+\lbrack30\mathrm{STS}{(\mathrm{reps})}^\ast0.34668\rbrack$$

**Fig. 1:**
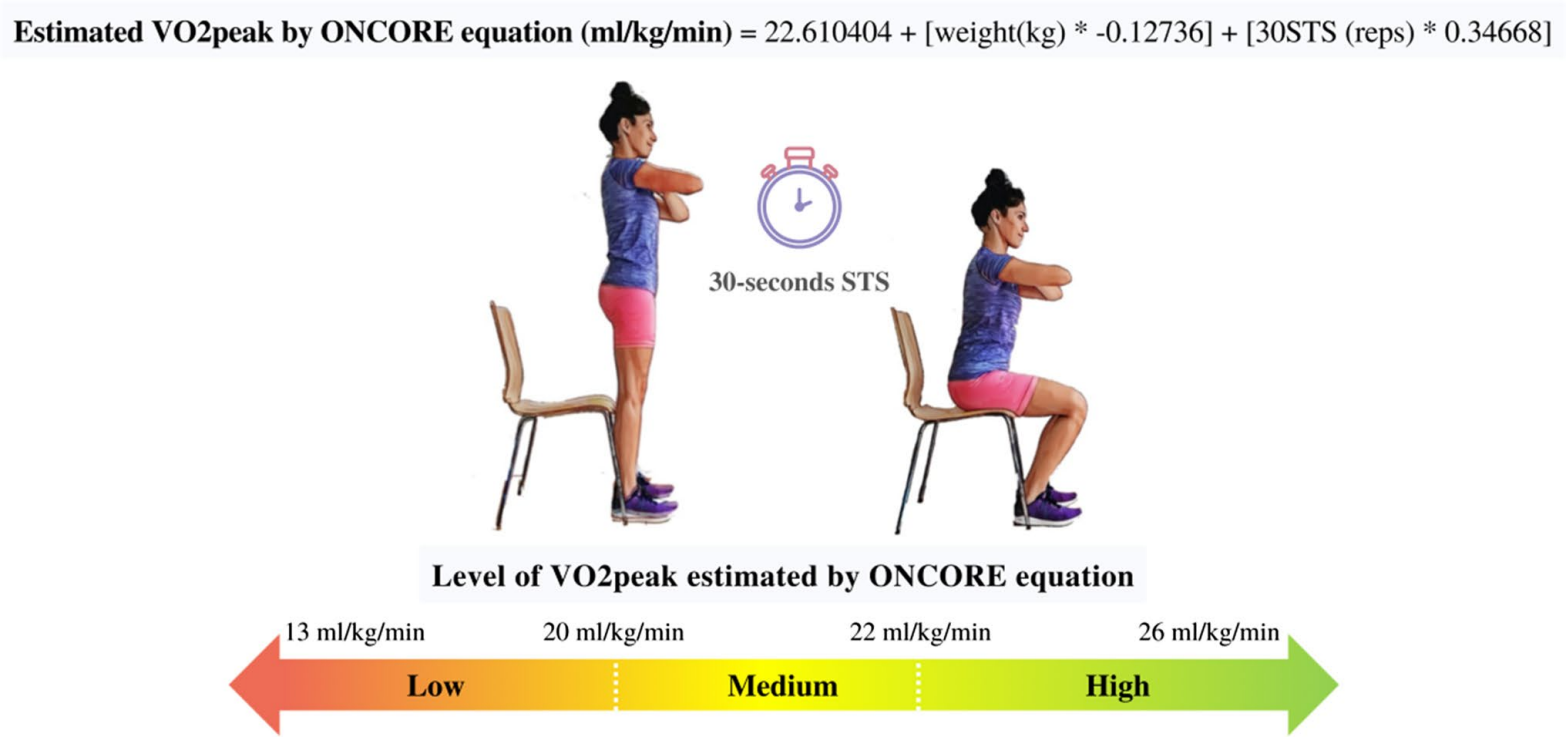
*Estimated VO2peak* with ONCORE equation from repetitions of the 30STS test in breast cancer women

Using the ONCORE equation, this new variable “Estimated VO2peak” was calculated for the whole sample of participants (*n* = 126), and the results were then stratified into tertiles. Three ranges of cardiopulmonary function were distinguished as low, medium, and high, delimited by the minimum value obtained (12.40 ml/kg/min): the 33rd percentile (20.45 ml/kg/min), the 66th percentile (21.91 ml/kg/min), and the maximum value (25.51 ml/kg/min). Figure 1 displays the estimation and stratification of VO2peak by the ONCORE equation, with slightly rounded values, the cut-off points being 13, 20, 22, and 26 ml/kg/min.

### Estimation and stratification of 6MWD by the ONCORE equation

In addition, following regression model #3 (Table 3), “Estimated 6MWD” was obtained using the ONCORE equation, including number of repetitions in the 30STS test and age (in years), as:

$$Estimated\;6MWD\;(m)=614.854778+\lbrack\mathrm{age}\;{(\mathrm{years})}^\ast-2.238046\rbrack+\lbrack30\mathrm{STS}{(\mathrm{reps})}^\ast5.149970\rbrack$$

Again, using the ONCORE equation, this new variable “Estimated 6MWD” was calculated for those patients who had performed the 6MWT (*n* = 85, pre and post), and the results were then stratified into tertiles. Three ranges of physical capacity were classified as low, medium, and high, delimited by the minimum value obtained (522.23 m): the 33rd percentile (606.05 m), the 66th percentile (627.95 m), and the maximum value (668.18 m).

## Discussion

To our knowledge, this is the first study to investigate the usefulness of the 30STS test for estimating VO2peak in women with breast cancer at early stages. Linear regression models were used to obtain the “Estimated VO2peak” and the “Estimated 6MWD” with an equation (called the ONCORE equation) based on the number of repetitions from the 30STS test. Using this tool could allow clinicians to obtain a reasonable *Estimated VO2peak* for assessing cardiopulmonary function in this population, by performing a simple 30STS test. Given the feasibility, briefness, and very low cost of the 30STS, it could be considered as the minimum outcome for evaluating cardiopulmonary function in women with breast cancer when other assessments are not available. Additionally, the stratification of patients into 3 levels of *Estimated* VO2peak (low, medium, and high) may facilitate screening and exercise prescription within oncological exercise programs. For this purpose, we have tried to transfer the knowledge into a free online calculator (ONCORE equation for *Estimated VO2peak: *http://ejercicioterapeuticouma.es/oncore-equation/) that could be useful in different clinical settings, including remote assessment for virtual exercise programs, increasingly common after the COVID-19 pandemic [29]. Furthermore, considering 6MWD is widely used to evaluate functional capacity, its estimation using the results of the 30STS test and age could also be useful. Therefore, the formula *Estimated 6MWD* by ONCORE equation could serve as a reference for clinicians who are more acquainted with this test.

This is the first time that the 30STS test is used for estimating VO2peak and/or 6MWD in women with breast cancer. Previous studies, such as the one conducted by Galiano et al. (2016) [17], have studied the relationship between the 10-repetitions STS and the 6MWT through non-parametric bilateral correlation, finding a weaker association (*r* =  − 0.283, *p* = 0.010), than the moderate parametric correlation obtained in our study (*r* = 0.440, *p* < 0.001). This difference may be due to the different nature of the STS test, while one records the time for 10-repetitions, the other collects the number of repetitions in 30 s. Nevertheless, the relationship between the 30STS test and other measurements of physical capacity has been analyzed in previous studies conducted in breast cancer patients, but those were the muscle strength, maximum power, lean body mass, or changes in self-reported fatigue [30, 31]

Several researchers have shown interest in the usefulness of the STS test as an alternative for estimating functional capacity in different pathologies and populations. Nakamura et al. (2019) observed a significant and very strong correlation (*r* = 0.89, *p* < 0.01) between VO2peak measured by an incremental STS test and VO2peak measured by CPET cycle ergometer in older patients with type 2 diabetes [32]. Melissa et al. (2017) analyzed the correlation between the 30STS test and the 6MWT in patients with head and neck cancer and observed a moderate association (*r* = 0.407) [33]. Likewise, recent investigations have tested the STS in chronic obstructive pulmonary disease (COPD) [24, 34]. The multicenter study by Crook et al. (2017) explored the physiological response to the 1-min STS test in COPD patients who participated in a pulmonary rehabilitation program in two centers (*n* = 52 and *n* = 203), finding a strong correlation between 1-min STS test and 6MWT at admission time (*r* = 0.59 and 0.64, respectively) and discharge (*r* = 0.67 and 0.68, respectively). In addition, a similar end-exercise physiological response (VO2, VCO2, RER, etc.) between both tests was observed [34]. Gregory et al. (2018) also concluded that the 1-min STS may be a good alternative to the 6MWT (*r* = 0.716, *p* < 0.001), with good reliability (ICC = 0.902) [24].

The applicability of the STS test in the assessment of certain patients with impaired global functional capacity is an important advantage and has been safely used. In breast cancer women, the STS test has been generally performed to evaluate lower limb strength, with number of repetitions being the main measurement [35, 36]. Cuesta et al. (2020) reported a mean value of 18,3 repetitions in breast cancer women [37], slightly lower than our sample, maybe due to differences in age, BMI, or the chair height which was 1 cm lower. The STS test has also been applied to evaluate the energy system (aerobic, anaerobic lactic, and alactic) in breast cancer women [37] and fatigue based on acceleration during the test [38].

In terms of VO2peak, mean relative value in our cohort (20.83 ± 4.26 ml/min/kg) is similar to that reported by other studies that performed the cycle ergometer test in woman with breast cancer, like the one by Oliver et al. (2014), with a mean value of 20.6 ± 6.7 ml/min/kg (*n* = 222) [39]. The review by Amanda et al. (2014) reported a slightly higher mean value of 24.6 ml/kg/min in women with breast cancer before adjuvant treatment. Nevertheless, this could be explained by the fact that most studies (16 of 27) used the treadmill walking test, which has been reported to lead to VO2peak values 10–20% higher compared to cycle ergometer [40]. The standard values for VO2peak derived from CPET using cycle ergometer are collected in the FRIEND Registry [41], from healthy men and women (aged 20–79), while no reference values are available to date for women with early stage breast cancer. It should be acknowledged that the mean value of our cohort corresponds to the 60th percentile of VO2peak in healthy women aged 40 to 49. As for 6MWT reference values, the systematic review and meta-analysis of But-Hadzic et al. (2021) reported a mean value of 477.4 m walked in breast cancer survivors [16], lower compared to our cohort (606.1 m), maybe because participants in our study, unlike those in the study by But-Hadzic et al., were at the beginning of treatment, with almost no side effects.

The main limitation of this study is that as it is a cross-sectional study, we cannot make predictions, but establish estimations based on the relationship between variables. A total of 126 measurements of VO2peak related to repetitions performed in the 30STS test have been recorded, which is a considerable number when studying this relationship for the first time. Second, it should be noted that the 30STS test requires a very short effort compared to the CPET, perhaps 1-min STS would have been more appropriate as it requires greater metabolic and higher hemodynamic demands [42, 43]. Main strengths of our research include direct measurement of VO2peak through CPET, the gold standard for assessing cardiopulmonary function. Also, we consider that the study, which was based on our own need to understand differences in cardiopulmonary function assessment and determine patient profiles to implement a CORe program, has a high potential for clinical application. Nevertheless, although alternatives to simplify the assessment are useful, CPET should not be disregarded as the gold standard, and should be made available to our patients whenever necessary.

## Conclusion

The present study showed that the 30STS test is a useful tool to estimate VO2peak in women with early stage breast cancer when CPET is not available. Our results showed moderate correlations between the 30STS test and VO2peak from CPET (*r* = 0.419), and between the 30STS test and 6MWD (*r* = 0.440). The ONCORE equations obtained from the multivariate regression models allow estimating of VO2peak and 6MWD based on a simple 30STS test. The stratification of the *Estimated VO2peak* and the *Estimated 6MWD* with ONCORE equations into three levels (low, moderate, and high) may facilitate the screening and monitoring of breast cancer patients for therapeutic exercise programs improving its efficiency.

## Data Availability

Data are available upon reasonable request.
